# Supplementary material for: Transcription factor Znf2 coordinates with the chromatin remodeling SWI/SNF complex to regulate cryptococcal cellular differentiation
Source: Commun Biol. 2019 Nov 14;2:412. doi: 10.1038/s42003-019-0665-2 (PMC6856107; doi:10.1038/s42003-019-0665-2)
Supplement: Supplementary file 1 — Supplementary Information [file 42003_2019_665_MOESM1_ESM.pdf]

Transcription factor Znf2 coordinates with the chromatin remodeling SWI/SNF complex to regulate cryptococcal cellular differentiation

Lin *et.al.*

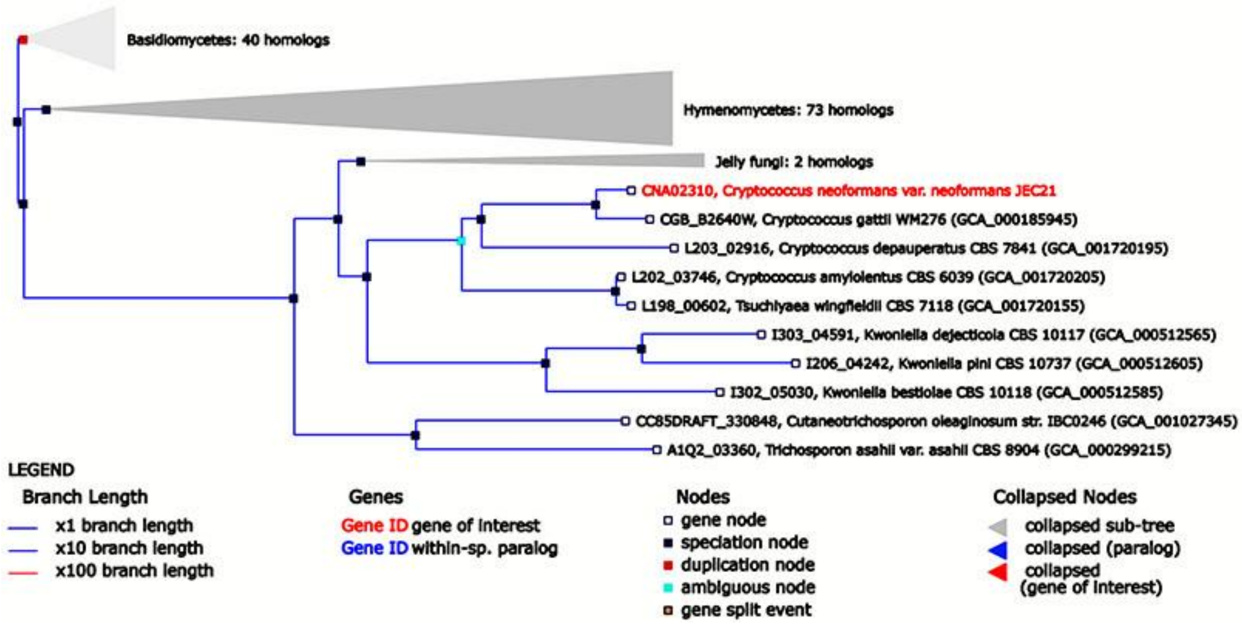

Supplementary Fig. 1. A *BRF1* gene tree. The gene tree was constructed on EnsemblFungi with the *BRF1* gene (gene ID *CNA02310*) as input. The gene tree was generated by the Gene Orthology/Paralogy prediction method pipeline where the maximum likelihood phylogenetic gene trees (generated by TreeBeST[5]) play a central role.

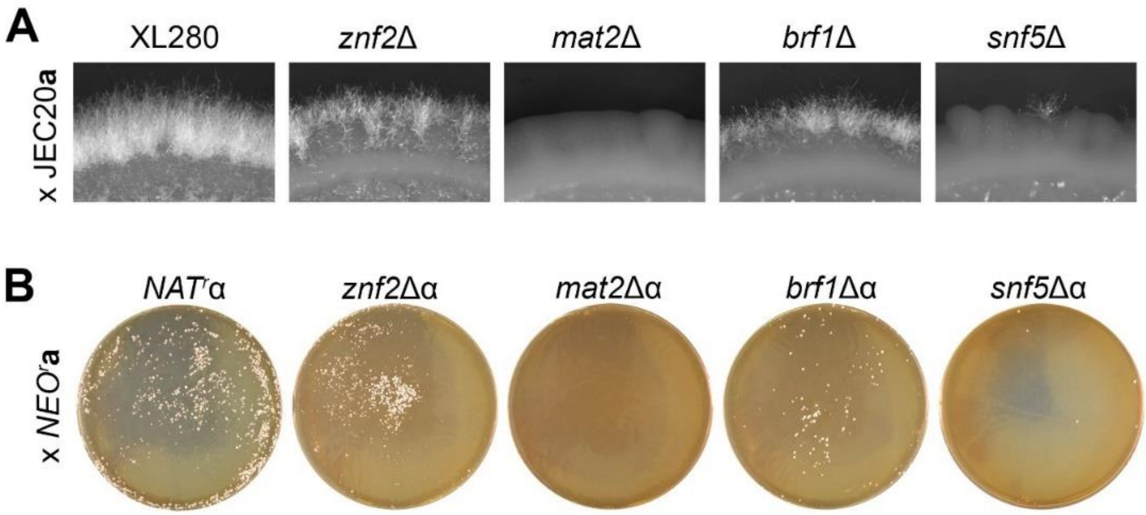

Supplementary Fig. 2. The *brf1*Δ and *snf5*Δ strains are defective in cell fusion. a. WT, *znf2*Δ, *mat2*Δ, *brf1*Δ, and *snf5*Δ α strains were crossed to the wild type JEC20**a** reference strain on V8 medium at 22°C in dark for 4 days. JEC20**a** is non-filamentous by itself on V8 medium. **b.** The α strains including the control XL1319, *znf2*Δ, *mat2*Δ, *brf1*Δ, and *snf5*Δ mutants with NAT^R were crossed with the mating type **a** strain YSB133 with G418^R. After 24 hours, the co-cultures were collected, and fusion products were selected on media supplemented with NAT and G418 drugs.

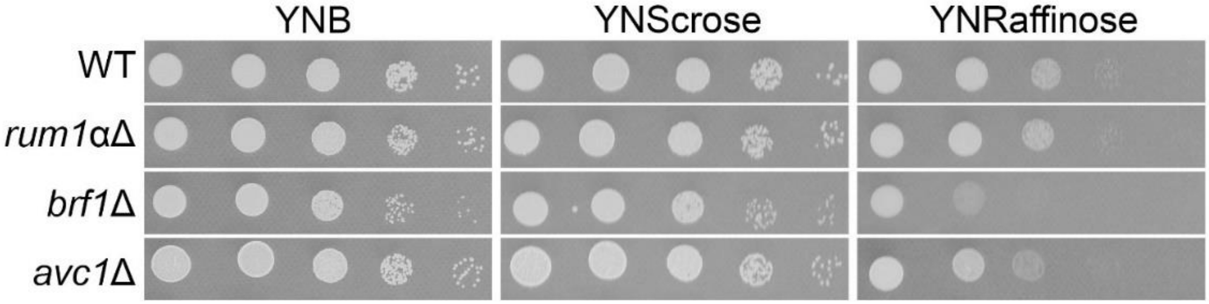

Supplementary Fig. 3. *BRF1* is the only ARID containing gene involved in growth on raffinose medium. The ARID containing gene deletion mutants *rum1α*Δ, *avc1*Δ and *brf1*Δ along with the WT XL280 were serial diluted and spotted onto YNB, YNSucrose, and YNRaffinose media and cultured at 30°C for 1 day.

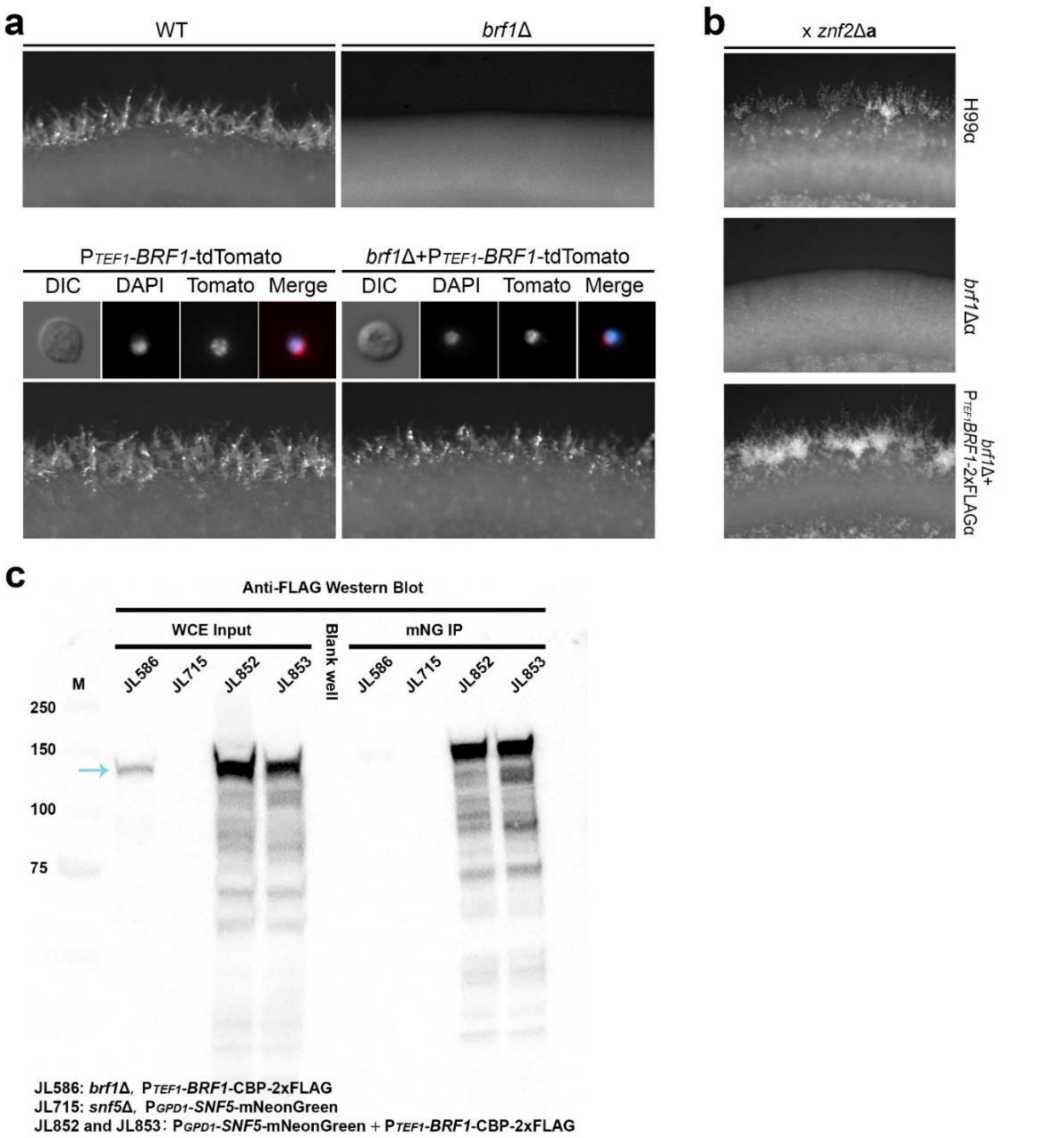

Supplementary Fig. 4. **The *BRF1* overexpression restored the defects in *brf1*Δ. a.** WT, *brf1*Δ, *brf1*Δ+P$_{TEF1}$-*BRF1*-tdTomato strains was cultured on V8 medium for 2 days. The fluorescence images were from the P$_{TEF1}$-*BRF1*-tdTomato cells cultured overnight in YPD liquid. **b.** WT H99, *brf1*Δ (JL131, MATα), and *brf1*Δ+P$_{TEF1}$-*BRF1*-CBP-2xFLAG strains were crossed with a *znf2*Δ strain (strain XT110, MAT**a**). The crosses were cultured on V8 medium for 3 days. **c.** Anti-FLAG Co-IP western of samples from JL586, JL715, JL852 and JL853. The IP samples (mNG IP, right) were elutes of the whole cell extract (WCE, left) incubated with mNeonGreen-Trap beads.

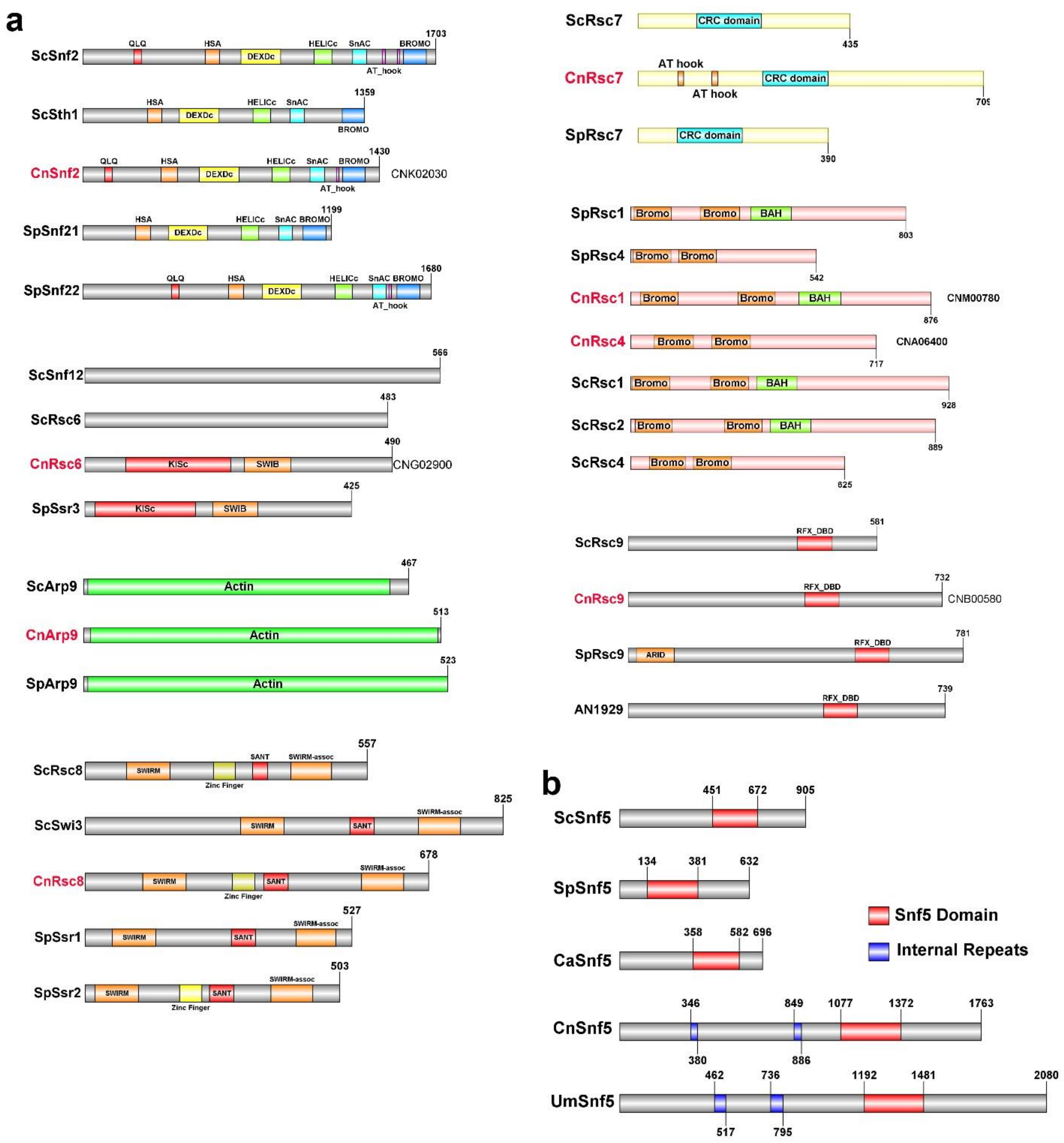

Supplementary Fig. 5. Diagrams of domain layout of the subunits in the SWI/SNF and RSC complex in *C. neoformans*. **a.** The domain layouts for Snf2, Rsc6, Arp9, Rsc8, Rsc7, Rsc1, Rsc4 and Rsc9 subunits in the RSC complex in *C. neoformans*, together with their homologs in *S. cerevisiae* and *S. pombe*. **b.** The domain layouts for Snf5 proteins in ascomycetes (*S. cerevisiae*, *S. pombe*, and *C. albicans*) and basidiomycetes (*C. neoformans* and *Ustilago maydis*).

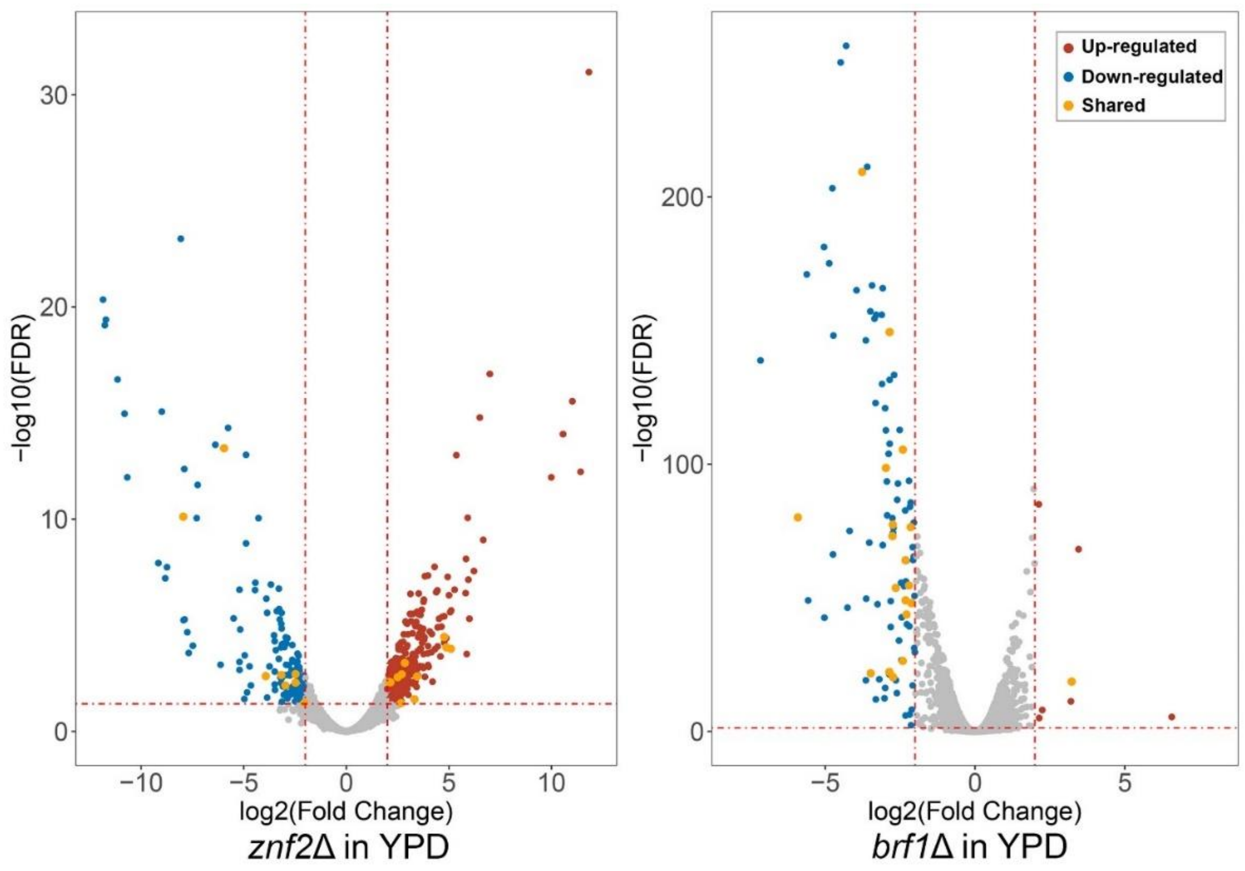

Supplementary Fig. 6. Volcano plots of fold changes in the gene transcript level in the *znf2*Δ mutant (left panel) and the *brf1*Δ mutant (right panel) compared to the wild type cultured in YPD medium for 24 hours. Each dot in the plot indicates a protein-coding gene. The vertical dash lines indicate the |$\log_2^{FC}$=2 and the horizontal dash line shows the FDR=0.05. The differentially up-regulated genes are colored red and the differentially down-regulated genes are colored blue. The shared DEG between *znf2*Δ and *brf1*Δ mutants are colored in orange.

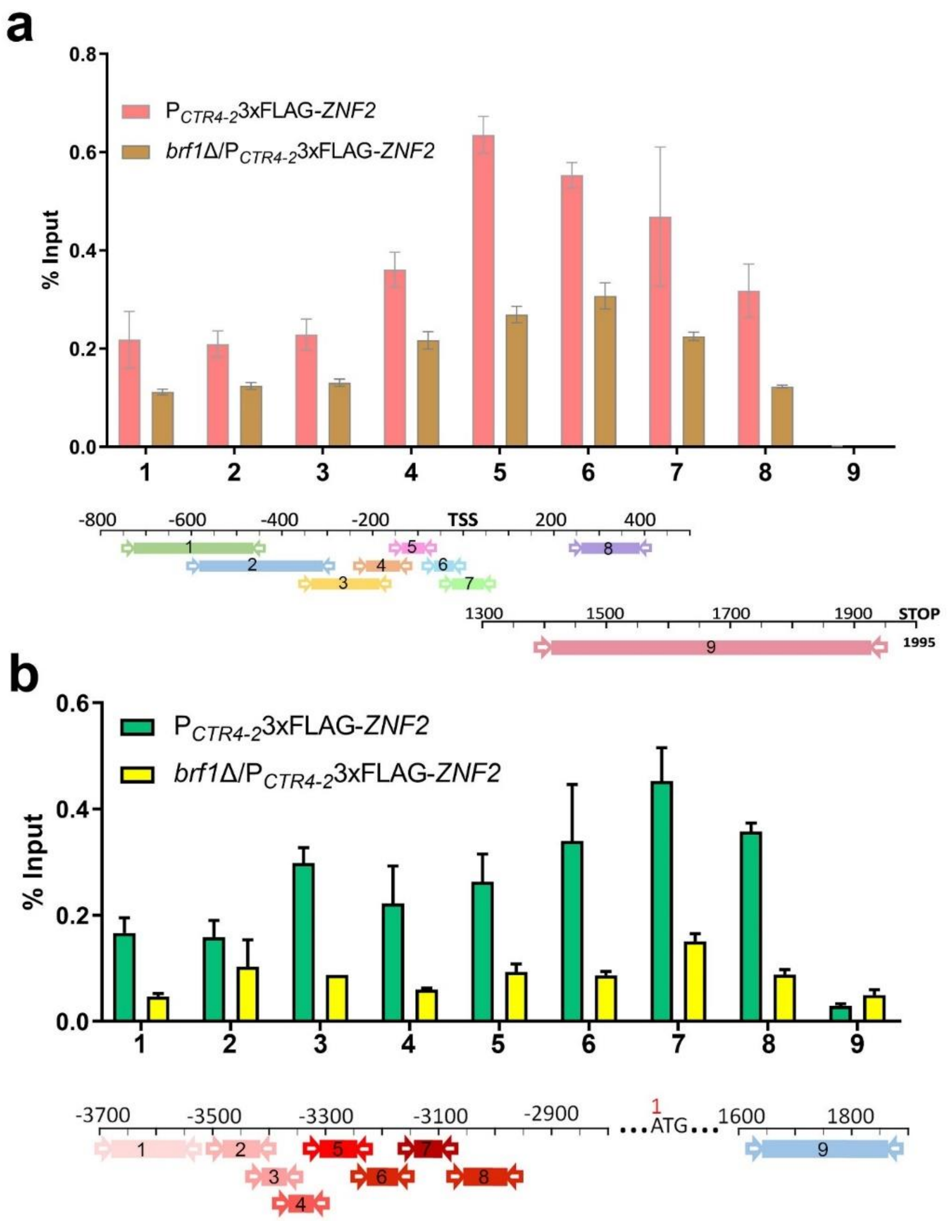

Supplementary Fig. 7. Znf2 binds to the promoter of *ZNF2* and *CFL1*. The %input bar graph indicates the relative abundance of DNA fragments of selected regions recovered from FLAG tagged Znf2 in the presence or absence of *BRF1*. The standard derivation error bars represent the data from two technical replicates of each biological duplicate. **a.** Primer pairs (from 1 to 8) spanned the promoter region of *CFL1*, and primer pair 9 covered the open reading frame and served as a control. The primer positions are relative to the transcription start site (TSS). **b.** Primer pairs (from 1 to 8) spanned the promoter region of *ZNF2*, and primer pair 9 covered the open reading frame and served as a control. The transcription start site of *ZNF2* gene is poorly defined. The primer positions are therefore relative to the translation start ATG site.

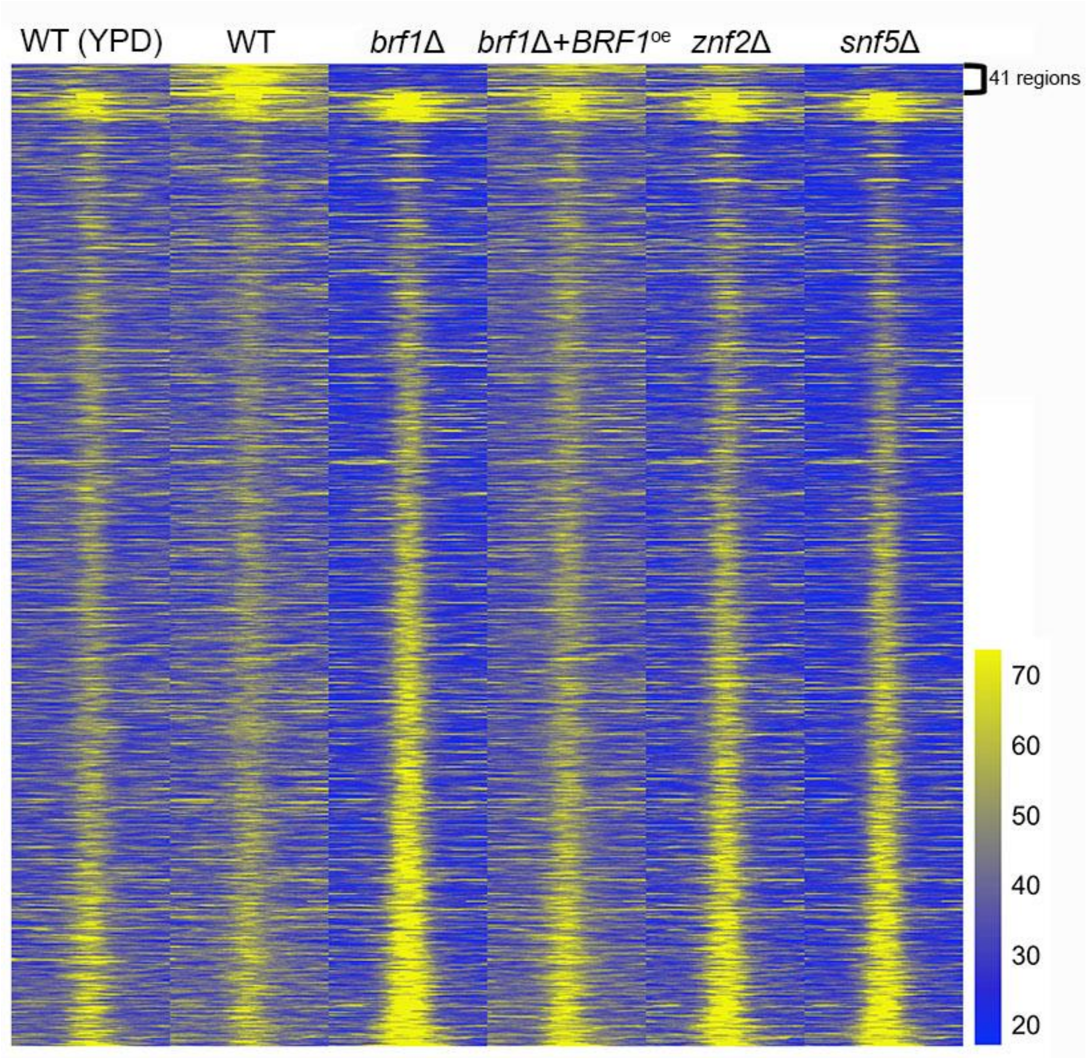

Supplementary Fig. 8. Heatmap of the *brf1*Δ differential peaks in WT, *brf1*Δ, *brf1*Δ+*BRF1*^{oe}, *znf2*Δ, and *snf5*Δ strains. Total of 1312 differential peaks were called by MACS2 using a q-value of 0.01, extension size of 73, and shifting reads by 37 bp to center on the insertion site in the *brf1*Δ compared to WT strain on V8 media. 41 of the regions of chromatin accessibility were reduced while the rest were enhanced. The chromatin accessibility of WT, *brf1*Δ, *brf1*Δ+*BRF1*^{oe}, *znf2*Δ, and *snf5*Δ strains were all mapped against the 1312 the *brf1*Δ differential peaks.

Supplementary Table 1. **related to Fig.1.** The list of insertion sites from the eight linked insertional mutants.

Type of Insertion	Insertion Site in the Genome	Potential Affected Gene(s)
Paired	169nt downstream of TSS	CNA02310
Paired	656nt downstream of TSS	CNA02310
Paired	2566nt downstream of TSS	CNA07190
Paired	126nt downstream of TSS	CNA07190
Paired	3068nt downstream of TSS	CND05760
Paired	1248nt downstream of TSS	CNK02410
Singleton	At intergenic region of CNA07150 and CNA07160	CNA07150|CNA07160
Singleton	At intergenic region of CNA07230 and CNA07240	CNA07230|CNA07240

Supplementary Table 2. **Related to Table 1 and Fig.3.** The list of proteins identified from Co-IP/MS by Sfh1-mNeonGreen as bait.

Coding Locus(D)	Coding Locus(A)	Protein name	# peptide spectrum matches
CNA03310	CNAG_00372	Rsc7 [c]	56
CNE04020	CNAG_02134	Rsc8 [b]	54
CNK02030	CNAG_01863	Snf2 [b]	50
**CNC06140**	**CNAG_03003**	**Sfh1** [c]	**35**
CNG02900	CNAG_03285	Rsc6 [b]	29
CNM00780	CNAG_06068	Rsc1 [c]	22
CNE02000	CNAG_02350	Rss1 [b]	22
CNB00580	CNAG_06744	Rsc9 [c]	18
CNB05320	CNAG_04048	Arp4 [b]	15
CNI00980	CNAG_04460	Arp9 [b]	12

[b]: the SWI/SNF and RSC complex shared subunits; [c]: the RSC-specific subunits.

Supplementary Table 3. **Related to Fig. 3.** The Snf5 domain search in fungi.

Kingdom	class	species	strain	#
Fungi	*Agaricomycetes*	*Coprinopsis cinerea*	okayama7#130	2
		Phanerochaete chrysosporium	RP-78	2
	Blastocladiomycetes	*Allomyces macrogynus*	ATCC 38327	4
	Chytridiomycetes	*Batrachochytrium dendrobatidis*	JEL423	3
		Spizellomyces punctatus	DAOM BR117	2
	Dothideomycetes	*Zymoseptoria tritici*	IPO323	2
	Eurotiomycetes	*Aspergillus aculeatus*	ATCC 16872	2
		Aspergillus brasiliensis	CBS 101740	2
		Aspergillus campestris	IBT 28561	2
		Aspergillus carbonarius	ITEM 5010	3
		Aspergillus clavatus	NRRL 1	2
		Aspergillus fischeri	NRRL 181	2
		Aspergillus flavus	NRRL3357	2
		Aspergillus fumigatus	A1163	2
			Af293	2
		Aspergillus glaucus	CBS 516.65	2
		Aspergillus kawachii	IFO 4308	2
		Aspergillus luchuensis	CBS 106.47	2
		Aspergillus nidulans	FGSC A4	2
		Aspergillus niger	ATCC 1015	2
			CBS 513.88	2
			strain N402 (ATCC64974)	2
		Aspergillus novofumigatus	IBT 16806	2
		Aspergillus ochraceoroseus	IBT 24754	2
		Aspergillus oryzae	RIB40	1
		Aspergillus steynii	IBT 23096	2
		Aspergillus sydowii	CBS 593.65	2
		Aspergillus terreus	NIH2624	2
		Aspergillus tubingensis	CBS 134.48	2
		Aspergillus versicolor	CBS 583.65	2
		Aspergillus wentii	DTO 134E9	2
		Aspergillus zonatus	CBS 506.65	2
		Coccidioides immitis	H538.4	2
			RS	2
		Coccidioides posadasii	C735 delta SOWgp	2
			RMSCC 3488	2
			str. Silveira	2
		Histoplasma capsulatum	G186AR	2
			G217B	2
			H143	2
			H88	2
			NAm1	2
		Paracoccidioides brasiliensis	Pb03	2
			Pb18	2
		Paracoccidioides lutzii	Pb01	2
		Penicillium rubens	Wisconsin 54-1255	2
		Talaromyces marneffei	ATCC 18224	2
		Talaromyces stipitatus	ATCC 10500	2
		Uncinocarpus reesii	1704	2
	Leotiomycetes	*Botrytis cinerea*	B05.10	2
		Sclerotinia sclerotiorum	1980 UF-70	2
	Pneumocystidomycetes	*Pneumocystis jirovecii*	SE8	2
	Pucciniomycetes	*Melampsora larici*		2
		Puccinia graminis	f. sp. tritici CRL 75-36-	2
	Saccharomycetes	*Candida albicans*	SC5314_B	2
			SC5314	2

			WO-1	2
		Candida auris	strain B8441	2
		Candida glabrata	CBS 138	3
		Candida parapsilosis	CDC317	2
		Clavispora lusitaniae	ATCC 42720	2
		Saccharomyces cerevisiae	S288c	2
		Yarrowia lipolytica	CLIB122	2
			CLIB89 (W29)	2
	Schizosaccharomycetes	*Schizosaccharomyces japonicus*	yFS275	2
		Schizosaccharomyces octosporus	yFS286	2
		Schizosaccharomyces pombe	972h-	2
	Sordariomycetes	*Fusarium fujikuroi*	IMI 58289	2
		Fusarium graminearum	PH-1	2
		Fusarium oxysporum	f. sp. melonis 26406	2
			f. sp. lycopersici 4287	2
			f. sp. cubense tropical race	2
			Fo47	2
			f. sp. cubense race 1	2
			f. sp. cubense race 4	2
		Fusarium proliferatum	ET1	2
		Fusarium verticillioides	7600	2
		Lomentospora prolificans	JHH-5317	2
		Magnaporthe oryzae	70-15	2
			BR32	2
		Neurospora crassa	OR74A	2
		Neurospora discreta	FGSC 8579	2
		Neurospora tetrasperma	FGSC 2508	2
		Scedosporium apiospermum	IHEM 14462	2
		Sordaria macrospora	k-hell	2
		Sporothrix brasiliensis	5110	2
		Sporothrix schenckii	1099-18	2
		Trichoderma reesei	QM6a	2
	Tremellomycetes	*Cryptococcus deuterogattii*	R265	2
		Cryptococcus gattii	CA1873	2
			EJB2	2
			VGIV IND107	2
			NT-10	2
			WM276	2
		Cryptococcus neoformans	var. neoformans B-3501A	2
			var. grubii H99	2
			var. neoformans JEC21	2
			var. grubii KN99	2
		Tremella mesenterica	DSM 1558	2
	Ustilaginomycetes	*Malassezia globosa*	CBS 7966	2
		Sporisorium reilianum	SRZ2	2
		Ustilago maydis	521	2
	Zygomycetes	*Mucor circinelloides*	f. lusitanicus CBS 277.49	3
		Phycomyces blakesleeanus	NRRL 1555(-)	3
		Rhizopus delemar	RA 99-880	1

Supplementary Table 4. The list of Snf2 proteins identified in selected fungi by RBOMO and BSA domains search in FungiDB.

**Phylum**	**Species**	**Gene locus**	**Product description**	**Strain background**
Basidiomycota	*Coprinopsis cinerea*	CC1G_08634	Snf2-family ATP dependent chromatin remodeling factor snf21	*C. cinerea okayama7#130*
	Puccinia graminis	PGTG_08738	Snf2-family ATP-dependent chromatin remodeling factor	*P. graminis f. sp. tritici CRL 75-36-700-3*
	Cryptococcus neoformans	CNK02030	Snf2 family, chromatin remodeling factor Snf2	*C. neoformans var. neoformans JEC21*
	Ustilago maydis	UMAG_11301	putative SWI/SNF catalytic subunit	*U. maydis 521*
Ascomycota	***Schizosaccharomyces pombe***	SPAC1250.01	RSC complex ATP-dependent DNA helicase **Snf21**	*S. pombe 972h-*
		SPCC1620.14c	SWI/SNF ATP-dependent DNA helicase **Snf22**	*S. pombe 972h-*
	Saccharomyces cerevisiae	YIL126W	RSC chromatin remodeling complex ATPase subunit **Sth1**	*S. cerevisiae S288c*
		YOR290C	SWI/SNF catalytic subunit **Snf2**	*S. cerevisiae S288c*
	Candida albicans	C2_02100W_A	Protein involved in transcriptional regulation; ortholog of *S. cerevisiae* Snf2p	*C. albicans SC5314*
		C3_02490C_A	Putative ATP-dependent helicase; induced by nitric oxide	*C. albicans SC5314*
	Candida glabrata	CAGL0G08756g	Ortholog(s) have DNA translocase activity, helicase activity, **Sth1**	*C. glabrata CBS 138*
		CAGL0M04807g	Catalytic subunit of the chromatin remodelling Swi/Snf complex, **Snf2**	*C. glabrata CBS 138*
	Aspergillus nidulans	AN2278	SNF2 family, chromatin remodeling factor	*A. nidulans FGSC A4*
	Fusarium oxysporum	FOZG_05129	SNF2-family ATP-dependent chromatin remodeling factor	*F. oxysporum Fo47*
	Sclerotinia sclerotiorum	SS1G_07017	SNF2-family ATP dependent chromatin remodeling factor	*S. sclerotiorum 1980 UF-70*
	Neurospora crassa	NCU06488	SNF2-family ATP dependent chromatin remodeling factor, crf3-1	*N. crassa OR74A*
Zygomycota	*Rhizopus delemar*	RO3G_14502	SNF2-family ATP dependent chromatin remodeling factor	*R. delemar RA 99-880*
	Phycomyces blakesleeanus	PHYBL_96129	Chromatin remodeling complex SWI/SNF, component SWI2 and related ATPases	*P. blakesleeanus NRRL 1555(-)*
	Mucor circinelloides	QYA_31459	Chromatin remodeling complex SWI/SNF, component SWI2 and related ATPases	*M. circinelloides f. lusitanicus CBS 277.49*
		QYA_136222	SNF2-family ATP dependent chromatin remodeling factor	*M. circinelloides f. lusitanicus CBS 277.49*

Supplementary Table 5. The strains used in this study.

Strain Name	Genotype	Sources
H99α	wild type	[1]
KN99**a**	wild type	[1]
XL280α	wild type	[2] , ATAC-seq, RNA-seq
XL280**a**	wild type	[3]
JEC21α	wild type	[4]
JEC20**a**	wild type	[4]
XX17	XL280α, *znf2*::NAT, $P_{CTR4\text{-}2}$-mCherry-*ZNF2*-NEO	[5], Insertional mutagenesis
XL574	XL280α, *znf2*::NAT	[6] , ATAC-seq, RNA-seq
XT110	XL280**a**, *znf2*::NAT	[7]
XL942	XL280α, *mat2*::NAT	[6]
XL1319	XL280α, *prf1*::NAT	[6]
YM61	XL280α, *rum1α* (*phd13*)::NAT	[8]
YSB133	KN99**a**, *can1*::NEO	[9]
LW377	XL280α, P_{GPD1}-*ZNF2*-V5 -NEO	
JL131	H99α, *brf1* (cnag_00240)::NAT	This study
JL233	XL280α, *brf1* (cna02310)::NAT	This study, ATAC-seq, RNA-seq
JL249	XL280**a**, *brf1*::NAT	This study
JL251	XL280α, *brf1*::NAT	This study
JL292	XL280α, *ssn6* (cnk02410)::NAT	This study
JL302	XL280α, *brf1*::NAT, P_{TEF1}-*BRF1*-tdTomato-NEO	This study
JL401	H99α, *brf1*::NAT, P_{TEF1}-*BRF1*-CBP-2xFLAG-NEO	This study, Co-IP/MS
JL402	H99α, *brf1*::NAT, P_{TEF1}-*BRF1*-CBP-2xFLAG-NEO	This study, Co-IP/MS
JL439	XL280**a**, *snf2* (cnk02030)::NAT	This study
JL440	XL280α, *snf2*::NAT	This study
JL444	XL280**a**, *snf5* (cna07190)::NAT	This study
JL445	XL280α, *snf5*::NAT	This study, ATAC-seq
JL486	XL280α, *sfh1* (cnc06140)::NAT	This study
JL552	XL280α, *rsc9* (cnb00580)::NAT	This study
JL555	XL280α, *avc1* (cnk00710)::NAT	This study
JL576	XL280α, *snf5*::NAT, *sfh1*::NAT	This study
JL586	XL280α, *brf1*::NAT, P_{TEF1}-*BRF1*-CBP-2xFLAG-NEO	This study, ATAC-seq, RNA-seq, Co-IP/western
JL589	XL280α, *sfh1*::NAT, P_{GPD1}-*SFH1*-mNeonGreen-NEO	This study, Co-IP/MS
JL596	XL280α, *brf1*::NAT, *snf5*::NEO	This study
JL650	XL280**a**, *znf2*::NAT, $P_{CTR4\text{-}2}$-3xFLAG-*ZNF2*-NEO	This study
JL653	XL280α, $P_{CTR4\text{-}2}$-3xFLAG-*ZNF2*-NEO	This study, ChIP-qPCR, ChIP-seq
JL655	XL280**a**, $P_{CTR4\text{-}2}$-3xFLAG-*ZNF2*-NEO	This study
JL665	XL280α, *brf1*::NAT, $P_{CTR4\text{-}2}$-3xFLAG-*ZNF2*-NEO	This study, ChIP-qPCR, ChIP-seq
JL715	XL280α, *snf5*::NAT, P_{GPD1}-*SNF5*-mNeonGreen-NEO,	Co-IP/western
JL852	XL280α, P_{GPD1}-*SNF5*-mNeonGreen-NEO, P_{TEF1}-*BRF1*-CBP-2xFLAG-NAT	Co-IP/western
JL853	XL280α, P_{GPD1}-*SNF5*-mNeonGreen-NEO, P_{TEF1}-*BRF1*-CBP-2xFLAG-NAT	Co-IP/western

Supplementary Table 6. The vectors used in this study.

**Vector Name**	**Genotype**	**Backbone**	**Sources**
pXL1	P_{GPD1}-Fse1-Pac1-T_{GPD1}-NEO	Topo2.1	
pXC	$P_{CTR4\text{-}2}$-Fse1-Pac1-T_{GPD1}-NEO	pXL1	
pYF5	P_{GPD1}-*PHD11*-mNeonGreen- T_{GPD1}-NEO	pUC19	This Study
pFZ3-*ZNF2*(D)	$P_{CTR4\text{-}2}$-3X FLAG-*ZNF2*(D)- T_{GPD1}-NEO	pUC19	This Study
pJL1	P_{TEF1}-Fse1-AsiS1-CBP-2X FLAG- T_{GPD1}-NEO	pXC	This Study
pJL2	P_{TEF1}-*BRF1*-CBP-2X FLAG- T_{GPD1}-NEO	pXL1	This Study
pJL3	P_{TEF1}-*BRF1*-tdTomato- T_{GPD1}-NEO	pXL1	This Study
pJL4	P_{GPD1}-*SFH1*-mNeonGreen- T_{GPD1}-NEO	pUC19	This Study
pJL5	P_{GPD1}-*SNF5*-mNeonGreen- T_{GPD1}-NEO	pUC19	This Study

Supplementary Table 7. The primers used in this study.

**Primer sequence**	**Annotation**	**Locus**
GTAAAACGACGGCCAG	M13F	
CAGGAAACAGCTATGAC	M13R	
TGTGGATGCTGGCGGAGGATA	P_{ACT1}-R	
AGGAGCATGATCCGATAC	*GPD1* terminator R	
TGCTTCTGTATTTGGGTTCTG	P_{CTR4-2} F	
GGCTCAAAGAGCAGATCAATG	U6 promoter Far-LF	U6
CCATCGATTTGCATTAGAACTAAAAACAAAGCA	U6 promoter F	U6
CCTCTGACACATGCAGCTCC	gRNA outside R	
CCGCTCGAGTAAAACAAAAAAGCACCGAC	gRNA-R	
TGATCTGGCCGGCCaaaaATGGACAAAAAATACAGC	FseI-Cas9 ORF F	
CTTGAAGTAGTCCTCCTTGAG	Cas9 screening R	
CCATAATGGACTTCCACCG	Far Left F	ZNF2
GGACAATCAGCTACCATCCA	LF	SNF5
ctggccgtcgttttacCTATAGGCGGACGTACAGTC	M13Fc+LR	SNF5
gtcatagctgtttcctgTCCACTGGTTCAACTGCAT	M13Rc+RF	SNF5
TATCATATGTGCAGTCATCTCAT	RR	SNF5
CTCCTGGTCGGCAGAGAT	Far Left F	SNF5
TGCTGAGTCTCATGCTATCTT	NEST R	SNF5
ATAACCACCAGTACGTCTCGG	Screen F	SNF5
AATATCAGATATGCGCTCACAA	Screen R	SNF5
TAATCTATAGCGTTCGCCGCAACAGTATACCCTGCCGGTG	gRNAc(snf5)-U6R	SNF5
GCGGCGAACGCTATAGATTAGTTTTAGAGCTAGAAATAGCAAGTT	gRNA(snf5)-ScaffoldF	SNF5
ATAggccggccATGAACAACCAAAACCAGCA	FseI+F	BRF1
ATAgcgatcgcTATCGAATCACCACCCCAG	AsiSI+prestopR	BRF1
CGGACTGCTTGAGAGCGA	LF	BRF1
ctggccgtcgttttacCAGAGTGCGACTAATGCGT	M13Fc+LR	BRF1
gtcatagctgtttcctgATCTGGGATGGGATTCGG	M13Rc+RF	BRF1
GCTTCTTGATATTGGCGACA	RR	BRF1
GTGCGATCTGAACTGCGG	Far Left F	BRF1
CAGCCGGCAGCAACAAC	NEST R	BRF1
GCTTGAGGATGCCCCG	NEST F	BRF1
GGTACTGGACAGGATCGGT	ORF-F	BRF1
CCTGCGGAACCAACCAA	Screen F/ ORF-mid-F	BRF1
TTATATCGAATCACCACCCC	Screen R	BRF1
TCAGCAGATCAATGACGTCCAACAGTATACCCTGCCGGTG	gRNAc(brf1)-U6R	BRF1
GGACGTCATTGATCTGCTGAGTTTTAGAGCTAGAAATAGCAAGTT	gRNA(brf1)-ScaffoldF	BRF1
ACGCTAACACTCAGGGTTGT	LF	SNF2
ctggccgtcgttttacCCACTTATCCGTCGTTCTTG	M13Fc+LR	SNF2
gtcatagctgtttcctgCTACGATACGTGACACCACAC	M13Rc+RF	SNF2
TGTAACTCTGGAGTCGGACTT	RR	SNF2
GAGTGAAGATCATCAGCCGT	Far Left F	SNF2
CGTCAGATGGTTACTCATTCG	NEST R	SNF2
CCGCATAGTAATCCACCTTG	ORF-R	SNF2
GGTGTGAAGAGACGCCAG	LF	SFH1
ctggccgtcgttttacTTGTCGATTACCCTGATTACG	M13Fc+LR	SFH1
gtcatagctgtttcctgCCAAGTTAGCTTCTGATCATTG	M13Rc+RF	SFH1
CAGGAGATTTGCGAGTGTAGTC	RR	SFH1
CTGTGAATGGTGACGAGCC	Far Left F	SFH1
GCTACATAACACGAATCACTCAG	NEST R	SFH1
CAGGCAGTGGAACGAGC	ORF F	SFH1
CAAGAGTTCTTCGGTGATGG	ORF R	SFH1
ATAggccggccCTCCCGTCTACGAAATGG	FseI+F	SFH1
ATTAgcgatcgcCCTTCTTCTCTTCAAAGGC	AsiSI+prestopR	SFH1
CAACTGACTTGCCTCTTTAGT	LF	RSC9
ctggccgtcgttttacCAAGAAGGGAAGAATGTGTG	M13Fc+LR	RSC9
gtcatagctgtttcctgGACATGTCTCAATTGGGTC	M13Rc+RF	RSC9

GAGAATGAGCGGATCGG	RR	RSC9
CGGGAAGATATCTCAGCC	Far Left F	RSC9
GGCTACTACAGACACTCGTC	NEST R	RSC9
CCTCATCATTGTCCAGTCC	ORF F	RSC9
GATGGTAGTTGCTAATGTGTTC	ORF R	RSC9
ACTCTGCTTTCTACAGCCCAAACAGTATACCCTGCCGGTG	gRNAc(rsc9)-U6R	RSC9
TGGGCTGTAGAAAGCAGAGTGTTTTAGAGCTAGAAATAGCAAGTT	gRNA(rsc9)-ScaffoldF	RSC9
TGTAAGTGAGCAGATTGTCCTG	LF	AVC1
ctggccgtcgttttacTATGTGGTGGCGTTCAATC	M13Fc+LR	AVC1
gtcatagctgtttcctgGAGGGCTTTGGCTAGCG	M13Rc+RF	AVC1
GTGGTCTATCACAGTTCGTCG	RR	AVC1
TCGGTGGAGCGGAAGTG	Far Left F	AVC1
GATGGAAGCCACCTCTGTG	NEST R	AVC1
CTACAGATGGAGCACAGCC	ORF F	AVC1
CATCGCAAGTACCTACCATG	ORF R	AVC1
TGTAAGTCCACGAGGTCATCAACAGTATACCCTGCCGGTG	gRNAc(avc1)-U6R	AVC1
GATGACCTCGTGGACTTACAGTTTTAGAGCTAGAAATAGCAAGTT	gRNA(avc1)-ScaffoldF	AVC1
GTCGTCGCACATCAGATGTC	LF	SSN6
ctggccgtcgttttacTGCTTCCAGCGTCATCG	M13Fc+LR	SSN6
gtcatagctgtttcctgAGGATGGACGAATGTGAGG	M13Rc+RF	SSN6
CTTGGCTGCTTACGATACG	RR	SSN6
TATTCCGTATTCCGTCATGA	Far Left F	SSN6
CGACGGAATATGCAATGCT	NEST R	SSN6
AATTGACGTTCACCCATCTC	Screen F/ ORF F	SSN6
GGAGTTGATGTCTGAGCAGTAC	Screen R/ ORF R	SSN6
AGTATCTTTATCCGACGCCCAACAGTATACCCTGCCGGTG	gRNA(ssn6)-U6R	SSN6
GGGCGTCGGATAAAGATACTGTTTTAGAGCTAGAAATAGCAAGTT	gRNA(ssn6)-ScaffoldF	SSN6
GCCCGAAGAAGCGGAATACA	1F	ZNF2
CTTTGGGCAATGTGCCTGAC	1R	ZNF2
ATTATGCCTGCTGCTCTGCT	2F	ZNF2
CCGGTCTCAAAGTACCGGAT	2R	ZNF2
CATGCTTGTGGCGTTCAGTT	3F	ZNF2
GTGTAGCTTGCAACTTCCGT	3R	ZNF2
CCGGTACTTTGAGACCGGAT	4F	ZNF2
TGCCTGCCACATCATTGAGA	4R	ZNF2
ACACTACTACTGTGGGCAGG	5F	ZNF2
CGTGCCAGCACTTCTCTTCT	5R	ZNF2
GGCTTGCATATGTCCGCAAT	6F	ZNF2
AACGCGTTATCTGGGTGCTT	6R	ZNF2
GAACCCAACGATGGAGACGG	7F	ZNF2
TAAGGTGAACACGAAGCGCC	7R	ZNF2
GCGCTTCGTGTTCACCTTAC	8F	ZNF2
CAGAGGACGATCAAGGGCTC	8R	ZNF2
TGGACATAGGAACGCTGACAAT	9F	ZNF2
GAACCTCGTTATGAGGCTTCTC	9R	ZNF2
GGCGCGTTGCTTCTTTTGAT	1F	CFL1
TCAGCTGTTCGCTGTCTGTC	1R	CFL1
GCATGGCTAGGGGTCAAAGT	2F	CFL1
ACAACAACGGTGATGGCTCT	2R	CFL1
TCAAGCTTCGTTTCAGCAGC	3F	CFL1
AATCTCCGGACGCAGGATTC	3R	CFL1
GCTGTCCATTGTCCATGAGC	4F	CFL1
TCACCCTACGGTTCCTGTCT	4R	CFL1
GCGTCCGGAGATTCTCATGT	5F	CFL1
ACCTCTTTGGGACGCGTATG	5R	CFL1
GATGCATACGCGTCCCAAAG	6F	CFL1
GGCGTGTTGTGATTGTGAGG	6R	CFL1
CCGAGCGCAAAGATGGATGA	7F	CFL1

TGTCCCGAGGATCTGATGGT	7R	CFL1
AGCTGCCTTGTCTTGCTTTCG	8F	CFL1
CCTAATAGCGCACTGGCAACC	8R	CFL1
CTCCACTCTCGTGCTCCTGAA	9F	CFL1
CCAGATTTGCAGCTGTAGAC	9R	CFL1

1 Nielsen, K. *et al.* Sexual cycle of *Cryptococcus neoformans var. grubii* and virulence of congenic a and alpha isolates. *Infection and immunity* **71**, 4831-4841 (2003).

2 Lin, X. R., Hull, C. M. & Heitman, J. Sexual reproduction between partners of the same mating type in *Cryptococcus neoformans*. *Nature* **434**, 1017-1021, doi:10.1038/nature03448 (2005).

3 Zhai, B. *et al.* Congenic strains of the filamentous form of *Cryptococcus neoformans* for studies of fungal morphogenesis and virulence. *Infection and immunity* **81**, 2626-2637, doi:10.1128/IAI.00259-13 (2013).

4 Kwon-Chung, K. J., Edman, J. C. & Wickes, B. L. Genetic association of mating types and virulence in *Cryptococcus neoformans*. *Infection and immunity* **60**, 602-605 (1992).

5 Wang, L., Zhai, B. & Lin, X. The link between morphotype transition and virulence in *Cryptococcus neoformans*. *PLoS pathogens* **8**, e1002765, doi:10.1371/journal.ppat.1002765 (2012).

6 Lin, X., Jackson, J. C., Feretzaki, M., Xue, C. & Heitman, J. Transcription factors Mat2 and Znf2 operate cellular circuits orchestrating opposite- and same-sex mating in *Cryptococcus neoformans*. *PLoS genetics* **6**, e1000953, doi:10.1371/journal.pgen.1000953 (2010).

7 Wang, L., Tian, X., Gyawali, R. & Lin, X. Fungal adhesion protein guides community behaviors and autoinduction in a paracrine manner. *Proceedings of the National Academy of Sciences of the United States of America* **110**, 11571-11576, doi:10.1073/pnas.1308173110 (2013).

8 Meng, Y. F., Fan, Y. M., Liao, W. Q. & Lin, X. R. Plant Homeodomain Genes Play Important Roles in Cryptococcal Yeast-Hypha Transition. *Applied and Environmental Microbiology* **84**, doi:UNSP e01732-1710.1128/AEM.01732-17 (2018).

9 Bahn, Y. S., Cox, G. M., Perfect, J. R. & Heitman, J. Carbonic anhydrase and CO2 sensing during *Cryptococcus neoformans* growth, differentiation, and virulence. *Curr Biol* **15**, 2013-2020, doi:10.1016/j.cub.2005.09.047 (2005).
