# Supplementary material for: Transcription factor Znf2 coordinates with the chromatin remodeling SWI/SNF complex to regulate cryptococcal cellular differentiation
Source: Commun Biol. 2019 Nov 14;2:412. doi: 10.1038/s42003-019-0665-2 (PMC6856107; doi:10.1038/s42003-019-0665-2)
Supplement: Supplementary file 2 — Description of Additional Supplementary Files [file 42003_2019_665_MOESM2_ESM.docx]

**Transcription factor Znf2 coordinates with the chromatin remodeling SWI/SNF complex to regulate cryptococcal cellular differentiation**

Lin *et.al.*

**Supplementary Data 1, related to Fig. 1**. The list of eighty-four filament-defective *Agrobacterium*-mediated transformants, and the meiotic progeny of some AMT mutants. The phenotypes of the AMT mutants from phenotypic screens are included as well.

**Supplementary Data 2, related to Fig. 4 and Supplementary Fig. 6**. The differentially expressed gene lists from RNA-seq. This dataset includes the DEGs of WT cells in filamentation inducing condition compared to filamentation suppression condition, DEGs of *znf2*Δ, *brf1*Δ and *brf1*Δ+*BRF1*^oe^ strains compared to WT under both filamentation inducing and filamentation suppression condition.

**Supplementary Data 3, related to Fig. 5.** The list of regions that are potentially bound by Znf2-FLAG.

**Supplementary Data 4, related to Fig. 5.** The list of peaks identified from ATAC-seq. This data set includes all the peaks identified from ATAC-seq in WT, *znf2*Δ, *brf1*Δ, *snf5*Δ, *brf1*Δ+*BRF1*^oe^ and *znf2*Δ+*BRF1*^oe^ strains grown in filamentation inducing V8 condition. WT grown in filamentation suppressing YPD condition was also included.

**Supplementary Data 5, related to Fig. 5**. The list of differential peaks identified in *brf1*Δ compared to WT from ATAC-seq. The differential peaks in *brf1*Δ were further annotated in this list.

**Supplementary Data 6, related to Fig. 5**. The list of differential peaks in mutants compared to WT in ATAC-seq. The peaks from Supplementary Table 6 were further analyzed and differential peaks were called with diffbind using standard parameters as included in this dataset.
