# Supplementary material for: Transcription factor Znf2 coordinates with the chromatin remodeling SWI/SNF complex to regulate cryptococcal cellular differentiation
Source: Commun Biol. 2019 Nov 14;2:412. doi: 10.1038/s42003-019-0665-2 (PMC6856107; doi:10.1038/s42003-019-0665-2)
Supplement: Supplementary file 9 — Reporting Summary [file 42003_2019_665_MOESM9_ESM.pdf]

nature research

Corresponding author(s): Xiaorong Lin

Last updated by author(s): Oct 11, 2019

Reporting Summary

Nature Research wishes to improve the reproducibility of the work that we publish. This form provides structure for consistency and transparency in reporting. For further information on Nature Research policies, see Authors & Referees and the Editorial Policy Checklist.

Statistics

For all statistical analyses, confirm that the following items are present in the figure legend, table legend, main text, or Methods section.

n/a	Confirmed	
☐	☒	The exact sample size (*n*) for each experimental group/condition, given as a discrete number and unit of measurement
☐	☒	A statement on whether measurements were taken from distinct samples or whether the same sample was measured repeatedly
☒	☐	The statistical test(s) used AND whether they are one- or two-sided<br>*Only common tests should be described solely by name; describe more complex techniques in the Methods section.*
☒	☐	A description of all covariates tested
☒	☐	A description of any assumptions or corrections, such as tests of normality and adjustment for multiple comparisons
☒	☐	A full description of the statistical parameters including central tendency (e.g. means) or other basic estimates (e.g. regression coefficient) AND variation (e.g. standard deviation) or associated estimates of uncertainty (e.g. confidence intervals)
☒	☐	For null hypothesis testing, the test statistic (e.g. *F*, *t*, *r*) with confidence intervals, effect sizes, degrees of freedom and *P* value noted<br>*Give P values as exact values whenever suitable.*
☒	☐	For Bayesian analysis, information on the choice of priors and Markov chain Monte Carlo settings
☒	☐	For hierarchical and complex designs, identification of the appropriate level for tests and full reporting of outcomes
☒	☐	Estimates of effect sizes (e.g. Cohen's *d*, Pearson's *r*), indicating how they were calculated

Our web collection on statistics for biologists contains articles on many of the points above.

Software and code

Policy information about availability of computer code

Data collection	All software and analytic tools used are described in the manuscript. Codes are available upon request.
Data analysis	All software and analytic tools used are described in the manuscript.

For manuscripts utilizing custom algorithms or software that are central to the research but not yet described in published literature, software must be made available to editors/reviewers. We strongly encourage code deposition in a community repository (e.g. GitHub). See the Nature Research guidelines for submitting code & software for further information.

Data

Policy information about availability of data

All manuscripts must include a data availability statement. This statement should provide the following information, where applicable:

- Accession codes, unique identifiers, or web links for publicly available datasets
- A list of figures that have associated raw data
- A description of any restrictions on data availability

All data supporting the findings of the current study are available within the article and its Supplementary Information files or from the corresponding author upon request. All RNA-seq and DNA-seq data has been deposited in NCBI under the BioProject accession number PRJNA534125. ATAC-seq and ChIP-seq data are available at NCBI GEO database with access number GSE137248.

Field-specific reporting

Please select the one below that is the best fit for your research. If you are not sure, read the appropriate sections before making your selection.

☒ Life sciences ☐ Behavioural & social sciences ☐ Ecological, evolutionary & environmental sciences

For a reference copy of the document with all sections, see nature.com/documents/nr-reporting-summary-flat.pdf

Life sciences study design

All studies must disclose on these points even when the disclosure is negative.

Sample size	N/A
Data exclusions	N/A
Replication	N/A
Randomization	N/A
Blinding	N/A

Behavioural & social sciences study design

All studies must disclose on these points even when the disclosure is negative.

Study description	*Briefly describe the study type including whether data are quantitative, qualitative, or mixed-methods (e.g. qualitative cross-sectional, quantitative experimental, mixed-methods case study).*
Research sample	*State the research sample (e.g. Harvard university undergraduates, villagers in rural India) and provide relevant demographic information (e.g. age, sex) and indicate whether the sample is representative. Provide a rationale for the study sample chosen. For studies involving existing datasets, please describe the dataset and source.*
Sampling strategy	*Describe the sampling procedure (e.g. random, snowball, stratified, convenience). Describe the statistical methods that were used to predetermine sample size OR if no sample-size calculation was performed, describe how sample sizes were chosen and provide a rationale for why these sample sizes are sufficient. For qualitative data, please indicate whether data saturation was considered, and what criteria were used to decide that no further sampling was needed.*
Data collection	*Provide details about the data collection procedure, including the instruments or devices used to record the data (e.g. pen and paper, computer, eye tracker, video or audio equipment) whether anyone was present besides the participant(s) and the researcher, and whether the researcher was blind to experimental condition and/or the study hypothesis during data collection.*
Timing	*Indicate the start and stop dates of data collection. If there is a gap between collection periods, state the dates for each sample cohort.*
Data exclusions	*If no data were excluded from the analyses, state so OR if data were excluded, provide the exact number of exclusions and the rationale behind them, indicating whether exclusion criteria were pre-established.*
Non-participation	*State how many participants dropped out/declined participation and the reason(s) given OR provide response rate OR state that no participants dropped out/declined participation.*
Randomization	*If participants were not allocated into experimental groups, state so OR describe how participants were allocated to groups, and if allocation was not random, describe how covariates were controlled.*

Ecological, evolutionary & environmental sciences study design

All studies must disclose on these points even when the disclosure is negative.

Study description	*Briefly describe the study. For quantitative data include treatment factors and interactions, design structure (e.g. factorial, nested, hierarchical), nature and number of experimental units and replicates.*
Research sample	*Describe the research sample (e.g. a group of tagged Passer domesticus, all Stenocereus thurberi within Organ Pipe Cactus National Monument), and provide a rationale for the sample choice. When relevant, describe the organism taxa, source, sex, age range and any manipulations. State what population the sample is meant to represent when applicable. For studies involving existing datasets, describe the data and its source.*

Sampling strategy	*Note the sampling procedure. Describe the statistical methods that were used to predetermine sample size OR if no sample-size calculation was performed, describe how sample sizes were chosen and provide a rationale for why these sample sizes are sufficient.*
Data collection	*Describe the data collection procedure, including who recorded the data and how.*
Timing and spatial scale	*Indicate the start and stop dates of data collection, noting the frequency and periodicity of sampling and providing a rationale for these choices. If there is a gap between collection periods, state the dates for each sample cohort. Specify the spatial scale from which the data are taken*
Data exclusions	*If no data were excluded from the analyses, state so OR if data were excluded, describe the exclusions and the rationale behind them, indicating whether exclusion criteria were pre-established.*
Reproducibility	*Describe the measures taken to verify the reproducibility of experimental findings. For each experiment, note whether any attempts to repeat the experiment failed OR state that all attempts to repeat the experiment were successful.*
Randomization	*Describe how samples/organisms/participants were allocated into groups. If allocation was not random, describe how covariates were controlled. If this is not relevant to your study, explain why.*
Blinding	*Describe the extent of blinding used during data acquisition and analysis. If blinding was not possible, describe why OR explain why blinding was not relevant to your study.*

Did the study involve field work? ☐ Yes ☐ No

Field work, collection and transport

Field conditions	*Describe the study conditions for field work, providing relevant parameters (e.g. temperature, rainfall).*
Location	*State the location of the sampling or experiment, providing relevant parameters (e.g. latitude and longitude, elevation, water depth).*
Access and import/export	*Describe the efforts you have made to access habitats and to collect and import/export your samples in a responsible manner and in compliance with local, national and international laws, noting any permits that were obtained (give the name of the issuing authority, the date of issue, and any identifying information).*
Disturbance	*Describe any disturbance caused by the study and how it was minimized.*

Reporting for specific materials, systems and methods

We require information from authors about some types of materials, experimental systems and methods used in many studies. Here, indicate whether each material, system or method listed is relevant to your study. If you are not sure if a list item applies to your research, read the appropriate section before selecting a response.

Materials & experimental systems

n/a	Involved in the study	
☐	☒	Antibodies
☒	☐	Eukaryotic cell lines
☒	☐	Palaeontology
☒	☐	Animals and other organisms
☒	☐	Human research participants
☒	☐	Clinical data

Methods

n/a	Involved in the study	
☐	☒	ChIP-seq
☒	☐	Flow cytometry
☒	☐	MRI-based neuroimaging

Antibodies

Antibodies used	All information can be found in the method and material section in the manuscript.
Validation	The antibodies we used were commercially available and have been testified by many independent labs. These antibodies are commonly used in many labs in Cryptococcus research field as well. Importantly, we include positive and negative controls in our experiments.

Eukaryotic cell lines

Policy information about cell lines

Cell line source(s)	*State the source of each cell line used.*
Authentication	*Describe the authentication procedures for each cell line used OR declare that none of the cell lines used were authenticated.*

Mycoplasma contamination | *Confirm that all cell lines tested negative for mycoplasma contamination OR describe the results of the testing for mycoplasma contamination OR declare that the cell lines were not tested for mycoplasma contamination.*

Commonly misidentified lines (See ICLAC register) | *Name any commonly misidentified cell lines used in the study and provide a rationale for their use.*

Palaeontology

Specimen provenance | *Provide provenance information for specimens and describe permits that were obtained for the work (including the name of the issuing authority, the date of issue, and any identifying information).*

Specimen deposition | *Indicate where the specimens have been deposited to permit free access by other researchers.*

Dating methods | *If new dates are provided, describe how they were obtained (e.g. collection, storage, sample pretreatment and measurement), where they were obtained (i.e. lab name), the calibration program and the protocol for quality assurance OR state that no new dates are provided.*

☐ Tick this box to confirm that the raw and calibrated dates are available in the paper or in Supplementary Information.

Animals and other organisms

Policy information about studies involving animals; ARRIVE guidelines recommended for reporting animal research

Laboratory animals | *For laboratory animals, report species, strain, sex and age OR state that the study did not involve laboratory animals.*

Wild animals | *Provide details on animals observed in or captured in the field; report species, sex and age where possible. Describe how animals were caught and transported and what happened to captive animals after the study (if killed, explain why and describe method; if released, say where and when) OR state that the study did not involve wild animals.*

Field-collected samples | *For laboratory work with field-collected samples, describe all relevant parameters such as housing, maintenance, temperature, photoperiod and end-of-experiment protocol OR state that the study did not involve samples collected from the field.*

Ethics oversight | *Identify the organization(s) that approved or provided guidance on the study protocol, OR state that no ethical approval or guidance was required and explain why not.*

Note that full information on the approval of the study protocol must also be provided in the manuscript.

Human research participants

Policy information about studies involving human research participants

Population characteristics | *Describe the covariate-relevant population characteristics of the human research participants (e.g. age, gender, genotypic information, past and current diagnosis and treatment categories). If you filled out the behavioural & social sciences study design questions and have nothing to add here, write "See above."*

Recruitment | *Describe how participants were recruited. Outline any potential self-selection bias or other biases that may be present and how these are likely to impact results.*

Ethics oversight | *Identify the organization(s) that approved the study protocol.*

Note that full information on the approval of the study protocol must also be provided in the manuscript.

Clinical data

Policy information about clinical studies

All manuscripts should comply with the ICMJE guidelines for publication of clinical research and a completed CONSORT checklist must be included with all submissions.

Clinical trial registration | *Provide the trial registration number from ClinicalTrials.gov or an equivalent agency.*

Study protocol | *Note where the full trial protocol can be accessed OR if not available, explain why.*

Data collection | *Describe the settings and locales of data collection, noting the time periods of recruitment and data collection.*

Outcomes | *Describe how you pre-defined primary and secondary outcome measures and how you assessed these measures.*

ChIP-seq

Data deposition

☒ Confirm that both raw and final processed data have been deposited in a public database such as GEO.

☒ Confirm that you have deposited or provided access to graph files (e.g. BED files) for the called peaks.

Data access links
May remain private before publication.

https://www.ncbi.nlm.nih.gov/geo/query/acc.cgi?acc=GSE137248

Files in database submission

GSM4073877	ATAC WT YPD rep 1
GSM4073878	ATAC WT YPD rep2
GSM4073879	ATAC WT V8 rep 1
GSM4073880	ATAC WT V8 rep 2
GSM4073881	ATAC dBRF1 rep 1
GSM4073882	ATAC dBRF1 rep 2
GSM4073883	ATAC dBRF1 _ BRF1oe rep 1
GSM4073884	ATAC dBRF1 + BRF1oe rep 2
GSM4073885	ATAC dZNF2 rep 1
GSM4073886	ATAC dZNF2 rep 2
GSM4073887	ATAC SNF5 rep 1
GSM4073888	ATAC SNF5 rep 2
GSM4073889	ChIP ZNF2-FLAG rep 1
GSM4073890	CHIP ZNF2-FLAG rep 1 input
GSM4073891	CHIP ZNF2 FLAG rep 2
GSM4073892	CHIP ZNF2 FLAG rep 2 input
GSM4073893	CHIP dBRF1 ZNF-FLAG rep 1
GSM4073894	CHIP dBRF1 ZNF-FLAG rep 1 input
GSM4073895	CHIP dBRF1 ZNF-FLAG rep 2
GSM4073896	CHIP dBRF1 ZNF-FLAG rep 2 input

Genome browser session
(e.g. UCSC)

http://chromatinlab.genetics.uga.edu/Linlab/?data=Cryptococcus_JEC21

Methodology

Replicates

We did biological duplicates in ATAC-seq and ChIP-seq experiment.

Sequencing depth

Paired-end sequencing for each sample generated between 9 - 40 million mapped reads for each sample

Sorted110_28_ChIP_MATalphaCTR4_3flag_ZNF2D_NEO_FLAG_Rep1_S28_R1_001.fastq.gz.bam
35023882 + 0 in total (QC-passed reads + QC-failed reads)
0 + 0 secondary
0 + 0 supplementary
0 + 0 duplicates
15586735 + 0 mapped (44.50% : N/A)
35023882 + 0 paired in sequencing
17511941 + 0 read1
17511941 + 0 read2
12659588 + 0 properly paired (36.15% : N/A)
14351240 + 0 with itself and mate mapped
1235495 + 0 singletons (3.53% : N/A)
1515980 + 0 with mate mapped to a different chr
1377109 + 0 with mate mapped to a different chr (mapQ>=5)

Sorted110_29_ChIP_MATalphaCTR4_3flag_ZNF2D_NEO_FLAG_Rep2_S29_R1_001.fastq.gz.bam
80885504 + 0 in total (QC-passed reads + QC-failed reads)
0 + 0 secondary
0 + 0 supplementary
0 + 0 duplicates
40685455 + 0 mapped (50.30% : N/A)
80885504 + 0 paired in sequencing
40442752 + 0 read1
40442752 + 0 read2
32233284 + 0 properly paired (39.85% : N/A)
37492320 + 0 with itself and mate mapped
3193135 + 0 singletons (3.95% : N/A)
4745140 + 0 with mate mapped to a different chr
4300252 + 0 with mate mapped to a different chr (mapQ>=5)

Sorted110_30_ChIP_MATalphabrf1NATCTR4_3flag_ZNF2D_NEO_FLAG_Rep1_S30_R1_001.fastq.gz.bam

20659036 + 0 in total (QC-passed reads + QC-failed reads)
0 + 0 secondary
0 + 0 supplementary
0 + 0 duplicates
9199602 + 0 mapped (44.53% : N/A)
20659036 + 0 paired in sequencing
10329518 + 0 read1
10329518 + 0 read2
7903670 + 0 properly paired (38.26% : N/A)
8683020 + 0 with itself and mate mapped
516582 + 0 singletons (2.50% : N/A)
678964 + 0 with mate mapped to a different chr
621089 + 0 with mate mapped to a different chr (mapQ>=5)

Sorted110_31_ChIP_MATalphabrf1NATCTR4_3flag_ZNF2D_NEO_FLAG_Rep2_S31_R1_001.fastq.gz.bam
44552050 + 0 in total (QC-passed reads + QC-failed reads)
0 + 0 secondary
0 + 0 supplementary
0 + 0 duplicates
30639660 + 0 mapped (68.77% : N/A)
44552050 + 0 paired in sequencing
22276025 + 0 read1
22276025 + 0 read2
27254346 + 0 properly paired (61.17% : N/A)
29393836 + 0 with itself and mate mapped
1245824 + 0 singletons (2.80% : N/A)
1911710 + 0 with mate mapped to a different chr
1742394 + 0 with mate mapped to a different chr (mapQ>=5)

Sorted110_32_ChIP_MATalphaCTR4_3flag_ZNF2D_NEO_input_Rep1_S32_R1_001.fastq.gz.bam
36320388 + 0 in total (QC-passed reads + QC-failed reads)
0 + 0 secondary
0 + 0 supplementary
0 + 0 duplicates
29357303 + 0 mapped (80.83% : N/A)
36320388 + 0 paired in sequencing
18160194 + 0 read1
18160194 + 0 read2
28515428 + 0 properly paired (78.51% : N/A)
28754456 + 0 with itself and mate mapped
602847 + 0 singletons (1.66% : N/A)
192332 + 0 with mate mapped to a different chr
156088 + 0 with mate mapped to a different chr (mapQ>=5)

Sorted110_33_ChIP_MATalphaCTR4_3flag_ZNF2D_NEO_input_Rep2_S33_R1_001.fastq.gz.bam
35961874 + 0 in total (QC-passed reads + QC-failed reads)
0 + 0 secondary
0 + 0 supplementary
0 + 0 duplicates
29352926 + 0 mapped (81.62% : N/A)
35961874 + 0 paired in sequencing
17980937 + 0 read1
17980937 + 0 read2
28448564 + 0 properly paired (79.11% : N/A)
28764962 + 0 with itself and mate mapped
587964 + 0 singletons (1.63% : N/A)
259608 + 0 with mate mapped to a different chr
218788 + 0 with mate mapped to a different chr (mapQ>=5)
Sorted110_34_ChIP_MATalphabrf1NATCTR4_3flag_ZNF2D_NEO_input_Rep1_S34_R1_001.fastq.gz.bam

We believe the low mapping rate is due to polymorphisms between the XL280 strain and the JEC21 genome.

Antibodies

anti-FLAG M2 monoclonal antibodies on magnetic beads (#M8823, Sigma)

Peak calling parameters

findPeaks <BamFileContainingTrimmedReadsWithDuplicatesRemoved.bam> -style factor -L 2.5 -localSize 20000 -o <NameOfPeakFile.txt>

Data quality

Replicate experiments were first inspected in the genome browser (see link above), revealing highly similar enrichment profiles. Peak calling was performed for individual replicate experiments and for combined replicates. Valid peaks had an

	FDR threshold < 0.0001 and an enrichment value > 4-fold over input.
Software	Software: The findPeaks module of the HOMER (Hypergeometric Optimization of Motif EnRichment; http://homer.ucsd.edu/homer/) software suite was used to call peaks.

Flow Cytometry

Plots

Confirm that:

- [ ] The axis labels state the marker and fluorochrome used (e.g. CD4-FITC).
- [ ] The axis scales are clearly visible. Include numbers along axes only for bottom left plot of group (a 'group' is an analysis of identical markers).
- [ ] All plots are contour plots with outliers or pseudocolor plots.
- [ ] A numerical value for number of cells or percentage (with statistics) is provided.

Methodology

Sample preparation	*Describe the sample preparation, detailing the biological source of the cells and any tissue processing steps used.*
Instrument	*Identify the instrument used for data collection, specifying make and model number.*
Software	*Describe the software used to collect and analyze the flow cytometry data. For custom code that has been deposited into a community repository, provide accession details.*
Cell population abundance	*Describe the abundance of the relevant cell populations within post-sort fractions, providing details on the purity of the samples and how it was determined.*
Gating strategy	*Describe the gating strategy used for all relevant experiments, specifying the preliminary FSC/SSC gates of the starting cell population, indicating where boundaries between "positive" and "negative" staining cell populations are defined.*

- [ ] Tick this box to confirm that a figure exemplifying the gating strategy is provided in the Supplementary Information.

Magnetic resonance imaging

Experimental design

Design type	*Indicate task or resting state; event-related or block design.*
Design specifications	*Specify the number of blocks, trials or experimental units per session and/or subject, and specify the length of each trial or block (if trials are blocked) and interval between trials.*
Behavioral performance measures	*State number and/or type of variables recorded (e.g. correct button press, response time) and what statistics were used to establish that the subjects were performing the task as expected (e.g. mean, range, and/or standard deviation across subjects).*

Acquisition

Imaging type(s)	*Specify: functional, structural, diffusion, perfusion.*
Field strength	*Specify in Tesla*
Sequence & imaging parameters	*Specify the pulse sequence type (gradient echo, spin echo, etc.), imaging type (EPI, spiral, etc.), field of view, matrix size, slice thickness, orientation and TE/TR/flip angle.*
Area of acquisition	*State whether a whole brain scan was used OR define the area of acquisition, describing how the region was determined.*

Diffusion MRI ☐ Used ☐ Not used

Preprocessing

Preprocessing software	*Provide detail on software version and revision number and on specific parameters (model/functions, brain extraction, segmentation, smoothing kernel size, etc.).*
Normalization	*If data were normalized/standardized, describe the approach(es): specify linear or non-linear and define image types used for transformation OR indicate that data were not normalized and explain rationale for lack of normalization.*

Normalization template	*Describe the template used for normalization/transformation, specifying subject space or group standardized space (e.g. original Talairach, MNI305, ICBM152) OR indicate that the data were not normalized.*
Noise and artifact removal	*Describe your procedure(s) for artifact and structured noise removal, specifying motion parameters, tissue signals and physiological signals (heart rate, respiration).*
Volume censoring	*Define your software and/or method and criteria for volume censoring, and state the extent of such censoring.*

Statistical modeling & inference

Model type and settings	*Specify type (mass univariate, multivariate, RSA, predictive, etc.) and describe essential details of the model at the first and second levels (e.g. fixed, random or mixed effects; drift or auto-correlation).*
Effect(s) tested	*Define precise effect in terms of the task or stimulus conditions instead of psychological concepts and indicate whether ANOVA or factorial designs were used.*

Specify type of analysis: ☐ Whole brain ☐ ROI-based ☐ Both

Statistic type for inference (See Eklund et al. 2016)	*Specify voxel-wise or cluster-wise and report all relevant parameters for cluster-wise methods.*
Correction	*Describe the type of correction and how it is obtained for multiple comparisons (e.g. FWE, FDR, permutation or Monte Carlo).*

Models & analysis

n/a	Involved in the study
☐	☐ Functional and/or effective connectivity
☐	☐ Graph analysis
☐	☐ Multivariate modeling or predictive analysis

Functional and/or effective connectivity	*Report the measures of dependence used and the model details (e.g. Pearson correlation, partial correlation, mutual information).*
Graph analysis	*Report the dependent variable and connectivity measure, specifying weighted graph or binarized graph, subject- or group-level, and the global and/or node summaries used (e.g. clustering coefficient, efficiency, etc.).*
Multivariate modeling and predictive analysis	*Specify independent variables, features extraction and dimension reduction, model, training and evaluation metrics.*
